# Survival of Embryos and Fry of Sea Trout (*Salmo trutta* m*. trutta*) Growing from Eggs Exposed to Different Concentrations of Selenium during Egg Swelling

**DOI:** 10.3390/ani11102921

**Published:** 2021-10-09

**Authors:** Katarzyna Dziewulska, Lucyna Kirczuk, Robert Czerniawski, Monika Kowalska-Góralska

**Affiliations:** 1 Department of Hydrobiology, Institute of Biology, University of Szczecin, Felczaka 3c Street, 71-412 Szczecin, Poland; lucyna.kirczuk@usz.edu.pl (L.K.); robert.czerniawski@usz.edu.pl (R.C.); 2 Molecular Biology and Biotechnology Centre, University of Szczecin, Wąska 13 Street, 71-415 Szczecin, Poland; 3 Department of Limnology and Fishery, Faculty of Biology and Animal Science, Institute of Animal Breeding, Wrocław University of Environmental and Life Sciences, Chełmońskiego 38c Street, 51-630 Wrocław, Poland; monika.kowalska-goralska@upwr.edu.pl

**Keywords:** aquaculture, fish, immersion, supplementation, toxicity

## Abstract

**Simple Summary:**

Selenium-poor areas are defined in the Earth. Adequate selenium (Se) consumption enhances the health and growth of organisms, but overdose of it can be harmful and pathogenic. The objective of the study was to investigate Se supplementation effects in the non-feeding stages of fish. Fertilised eggs of sea trout were immersed in water enriched with Se during the egg-swelling period. Se at a concentration from 0.5 to 8 mg Se L^−1^ slightly impacted the hatching rate. A higher concentration of Se resulted in declined survival of embryos. Accumulation of Se in the body of hatch increased with the increase of element concentration in the aqueous environment during egg swelling. The survival of fry was similar in all groups, while the fry length and weight correlated positively with Se concentration in its body. In Se-poor areas, immersion of fertilised eggs in water enriched with Se during egg swelling can be used to supplement the deficient element to fish in order to improve breeding outcomes in aquaculture.

**Abstract:**

Adequate selenium (Se) availability enhances the health and growth of organisms, but overdose of it can be harmful and pathogenic. The study’s objective was to analyse the impact of short-term exposure of sea trout fertilised eggs to inorganic selenium (SeO_2_) at concentrations from 0 to 32 mg Se L^−1^ to find the optimal and toxic dose of Se on early fish development. Se accumulated in the body, embryos’ survival rate, and growth in the first four months of life was examined. Swelling of fertilised eggs in water supplemented with Se at a concentration from 0.5 to 8 mg Se L^−1^ was associated with a slightly positive impact on the hatching rate. At higher Se concentration, a harmful effect on the survival of the embryo was observed. The survival of fry was similar in all groups, while the fry length and weight correlated positively with Se concentration in its body. Immersion of fertilised eggs in water enriched with Se during egg swelling can constitute a method to supplement the element to non-feeding stages of fish. In selenium-poor areas, this innovative method can be implemented in aquaculture to improve breeding outcomes. Se concentration should be adjusted to the chemical compound, fish species, and Se’s content in the yolk.

## 1. Introduction

Selenium is a vital trace element incorporated into selenoproteins, which have an extensive impact on the organism. A suitable supply of selenium to the organism is vital for normal functioning [1,2]. The element supports muscle function, fertility and reproduction, metabolism, and DNA synthesis [3,4,5,6,7,8,9,10,11,12]. Selenium is a part of glutathione peroxidase (GPx), and thioredoxin reductase (TrxR) enzymes fulfilling a key role against oxidative cell injury and prevents the effects of exposing the animals to prolonged stress. The anticarcinogenic effect and antagonistic effect of selenium on heavy metals are also known [1,13,14,15,16,17,18,19]. Low selenium uptake results in a lower growth rate, weaker reproduction, impaired immune function, and increased mortality [12,14,19,20,21]. In animals and humans, selenium deficiency can trigger many diseases and disorders such as peripheral myopathy, necrotising cardiomyopathy, decreased muscle tone and conduction disturbances, hair thinning, opacification of the nails, and anaemia [16,17,22].

An excessive status of selenium in the body is toxic, resulting in impaired enzyme function and disruption of protein structure [23,24,25]. In regions contaminated by selenium, reduced fertility, damaged parenchymal organs, musculoskeletal disorders, teratogenicity, and lethality were found [26,27,28,29,30]. Younger forms are typically more sensitive to its harmful effects [31,32,33]. Embryos are particularly vulnerable to its impact because selenium particularly accumulates in the yolk of eggs, which they feed on [34,35,36,37].

In the environment, selenium is found in different oxidation states in organic and inorganic compounds. Inorganic forms as elemental selenium, selenite and selenides exhibit low uptake—organic forms of selenium, such as selenomethionine, selenocysteine, and methylselenocysteine exhibit higher bioavailability [19,38,39]. Generally, selenium concentration in water is below the limit sets by the United States Environmental Protection Agency (EPA) (1.5–3.1 µg L^−1^) [40] or World Health Organization (WHO) (40 µg L^−1^ for drinking water) [41] except in regions with rock and soil rich in selenium and anthropogenic contaminated areas. Anthropogenic sources of Se leading to environmental pollution mainly derive from mining, agriculture, oil refining, glass, and medical manufactures [42,43,44,45]. In the world, many areas are poor in selenium, which is linked to its low level in soil, water, and plant food [34,46,47,48]. Therefore, selenium supplementation of feed for domestic animals, including fish, is recommended [49,50,51,52,53]. Selenium is moved up the food chain. Excessive availability in the environment and nutrition is subject to bioaccumulation in living organisms and toxic [35,54]. The European Food Safety Authority (EFSA) [55] recommends supplementation levels of up to 0.2 mg kg^−1^ of organic selenium in feed to ensure consumer safety. In adult fish selenium requirement for optimal growth is higher [9,19,39,56,57,58].

Sea trout (*Salmo trutta* m. *trutta* L.) is a migrating salmonid fish. Adult fish leave marine waters and enter rivers to spawn. The natural range of occurrence of the species includes Atlantic, North, White, and Baltic Sea basins. In Poland, the sea trout is an economically important species due to the extinction of the Atlantic salmon in the 1980s [59]. Unfortunately, changes in hydrology resulting from logging and the development of agriculture, construction of rivers dams overfishing systematically diminished resources of the species. At the same time, actions have been undertaken to restore the resources of these fishes mainly by stocking. Stocking material is obtained by controlled reproduction using natural spawners and breeding offspring at a fish farm [60,61,62,63,64]. It is important to improve the conditions of controlled spawning and breeding in order to obtain high production of healthy stocking material. The Baltic Sea and Poland are selenium poor areas [46,65]. The mean selenium content in Polish waters is below 0.08 µg L^−1^ [66,67]. The swelling is the moment in which the chorion is permeable for water and the substances dissolved therein [68]. In aquaculture, selenium uptake during swelling can enrich the yolk with the element and improve the breeding effect’s hatching results. Selenium dioxide is the inorganic compound commonly used in the industry apart from another inorganic form as selenite and sodium selenate [17]. The study aimed to analyse the effect of supplementation of water with selenium dioxide used for egg swelling on the survival rate of individual embryonic stages and growth of sea trout to find the optimal and toxic dose of Se.

## 2. Materials and Methods

### 2.1. Experiment Description

Eggs and milt of wild sea trout (*Salmo trutta* m. *trutta* L.) entering the Rega River for spawning (NW Poland) were purchased from Polish Fishing Association division Szczecin. The eggs and milt originated from three individuals of each sex. After transport (4 °C) to the laboratory and assessing milt (using computer-assisted sperm analysis (CASA)) and eggs’ quality, the gametes were mixed in equal proportions from different individuals in order to obtain fertilisation. After rinsing the fertilised eggs with aquarium water from a closed-circuit, they were divided into eight groups of 150 eggs, each in three repetitions, each containing about 50 eggs. Se content in unfertilised egg and hatchery water was very low (selenium-poor area). In egg, the element concentration was 1.6 µg Se g^−1^ (dw) (assuming 65% moisture of eggs [69,70]) and in aquarium water of 0.04 µg Se L^−1^. Considering Se content in egg and water, envelope permeability, optimal demand for Se for young stages, and to detect toxic concentration for embryos, eight concentrations of selenium 0, 0.5, 1, 2, 4, 8, 16 and 32 mg Se L^−1^ (experimental groups 0, 0.5, 1, 2, 4, 8, 16, 32 Se) were prepared to dissolve SeO_2_ (Merck) in separated aquarium water. Fifty fertilised eggs were placed in 100 mL of selenium solution for 3 h to swell at 6 °C. After the swelling, the eggs were rinsed with separated aquarium water and transferred to baskets floating in the basins (three repetitions) with a closed water circuit. The survival rate was monitored by counting and removing white eggs. Hatching time was similar in all groups between February 18 and 27. Three weeks after hatching, fish from selected variants (0, 1, 2, 8, 16 Se) was reared in basins of 300 L to the age of four months. Initially, the water temperature was kept at 6 °C; then, it gradually increased in subsequent months to 16 °C. Water flow was 300 L h^−1^. Chemical and physical parameters of water were recorded three times a day. During rearing, N-NH_3_ mean concentration was 0.007 mg L^−1^ ± 0.003, diluted oxygen 7.20 mg L^−1^ ± 0.41, and pH 7.41 ± 0.82, photoperiod typical of this season. Fry was fed with frozen foods, adult *Daphni*a 1.13 µg Se g^−1^ (dw), *Chironomidae* larvae 0.29 µg Se g^−1^ (dw), and granulated feed (Skretting. Perla Larva Proaktive 4.0) containing 0.23 µg Se g^−1^ (dw). Feeding time takes place between 7.00 a.m.–5.00 p.m. every 3–4 h.

At monthly intervals from the beginning of feeding, 30 individuals were collected from each basin for morphometric testing from March through June. After short-term anaesthesia in MS-222, measurements of fish body weight (W) with accuracy to 0.1 mg and length (fork length, L_F_) were taken using callipers with an accuracy of 0.1 mm. After the measurements, fish were released to the basin. Condition factor CF was calculated using the following formula CF = 100 × W × L_F_^−3^. According to Polish law, permission from the Ethics Committee for Animal Experimentation (no. 86/2016) was obtained to conduct the experiment.

### 2.2. Determination of Selenium Accumulation in the Body of Hatching and Fry

The concentration of accumulated selenium was determined in wet weight (ww) in the hatch and four-month-old fry euthanised by MS-222 overdose and stunning with the destruction of the brain. For the purpose of mineralisation, 2 g of wet material was collected. The material was mineralised in a MARS-5 microwave oven by CAM (Matthews, USA). In order to check the accuracy of the method, selenium content was determined in a reference material—DOLT-2 from the National Research Council of Canada. Selenium reference content was 6060 ± 409 μg kg^−1^. The value obtained in the analytical procedure was 5850 ± 432 μg kg^−1^. Selenium concentrations were determined using hydride generation atomic absorption spectrometry (HG AAS) on a VARIAN SpectrAA 220 FS apparatus (Melbourne, Australia). The methodology of Diaz-Alarcon et al. [71] was utilised.

### 2.3. Statistical Analysis

Shapiro–Wilk test of normality of the analysed data and Levene variance homogeneity test were conducted. One-way ANOVAs was used to test the significance of differences in selenium content accumulated in hatching and four-month-old fry between the experimental groups. Two-way repeated-measures ANOVA was used to analyse the significance of differences in length, weight, condition factor, and intestinal and renal epithelial cell height between the experimental groups. Fisher’s least significant difference test was used for post hoc comparisons. A chi-squared test was used to compare the frequencies of fry survival. Pearson’s correlation coefficients were calculated to evaluate the relationship between selenium concentration in the fish body and fish length and weight. All analyses were performed at a significance level of 0.05 using the Statistica v. 13.1 software (StatSoft, Inc., Dell Inc., Tulsa, OK, USA). The data are presented as means ± SD.

## 3. Results

### 3.1. Survival of the Embryos until Hatching

**Figure 1:**
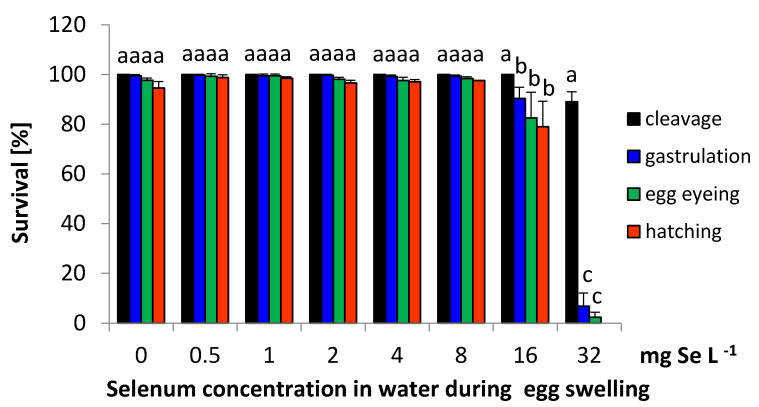
The survival rate of sea trout (*Salmo trutta* m*. trutta*) embryos grows from eggs exposed to different concentrations of inorganic selenium (SeO_2_) in water during egg swelling 0, 0.5, 1, 2, 4, 8, 16, and 32 mg Se L^−1^. Values marked with the same letter are not significantly different from one another (*p* > 0.05). Two-way ANOVAs and then Fisher tests were used for the post-hoc comparison.

Survival of sea trout embryos in the control group at the subsequent developmental stages: cleavage, gastrulation, eyed-egg, and hatching were 100 ± 0%, 99.5 ± 0.5%, 97.9 ± 0.9% and 95.3 ± 2.7%, respectively (Figure 1). In 0.5–1 and 2–8 Se variants the hatching rate (98.5–98.7% and 96.5–97.6%) was slightly higher than in control group (95.3%) (*p* > 0.05) (Figure 1). The increased embryo mortality rate was observed for the groups with higher selenium concentrations. In the 16 mg, Se L^−1^ group, significantly higher mortality was observed from the gastrulation stage (*p* < 0.05). The survival rate in this variant at cleavage, gastrulation, eyed-egg, and hatching stage was 100 ± 0%, 90.4 ± 4.5%, 82.5 ± 10.4%, and 79.0 ± 10.3%, respectively (Figure 1).

The highest selenium amount added to the water (32 mg Se L^−1^) was lethal for embryos. In this group, higher mortality of 11.0 ± 4.0% at the cleavage stage was detected (*p* < 0.05). During gastrulation, in this group, mortality was very high (mean 92.4 ± 5.7%); at eyed-egg, all of the eggs died (LT_100_) (Figure 1).

None of the macroscopically detected body deformations was found in hatched larvae in the control sample. In experimental groups, singular cases of spine deformities were observed; they comprised 0.3% of individuals in all experimental variants.

### 3.2. Selenium Content in the Body of Hatch and Fry

Selenium concentration accumulated in the hatch body increased with increasing the element concentration in the aqueous environment during egg swelling (Table 1). Detection of selenium content in hatch body revealed the lowest Se concentration in the control group, average Se status 3.6 ± 0.2 µg g^−1^ (dw), whereas, in the 16 Se group, selenium concentration was 5.4 ± 0.3 µg g^−1^ (*p* < 0.05) (Table 1).

Differences in selenium concentration in the fish body of the experimental group were maintained in four-month-old fry. In the control group, the mean concentration of selenium in the fish body was 2.7, while in the 16 Se group was 4.9 µg g^−1^ (dw) (*p* < 0.05) (Table 1).

### 3.3. Survival Rate, Growth and Condition of Fry during Rearing

**Figure 2:**
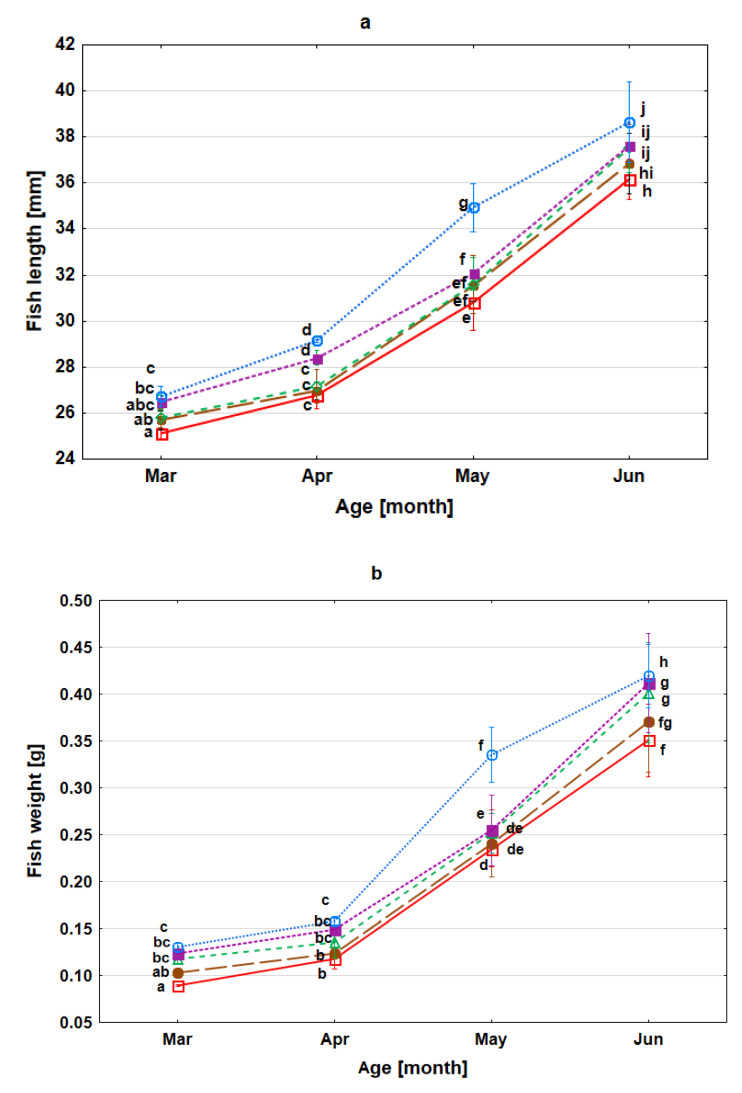
The Length (fork length) (**a**) and weight (**b**) of the sea trout fry (*Salmo trutta* m*. trutta*) developed from eggs exposed to different concentrations of inorganic selenium (SeO_2_) in water during egg swelling 0, 1, 2, 8, 16 mg Se L^−1^. Values marked with the same letter are not significantly different from one another (*p* > 0.05). Two-way ANOVAs and then Fisher tests were used for the post-hoc comparison. □– 0 Se (red); ●– 1 Se (brown); ∆– 2 Se (green); ■– 8 Se (violet); ○– 16 Se (blue).

The survival rate of fry to the age of four months did not differ between control and experimental samples; it remained in the range from 79.8 to 86.2% (*p* > 0.05), whereas the growth of fry differed between variants (*p* < 0.05). In the group of fish with higher selenium content in the body, greater length and weight were recorded (*p* < 0.05). The mean body length of one-month-old fry in the control group was lowest on average of 25.12 ± 0.57 mm. Statistically, significantly greater lengths were observed in samples 8 and 16 Se hatching, which attained 26.47 ± 0.81 mm and 26.73 ± 1.21 mm, respectively (Figure 2a). Similar differences were observed for body weight in one-month-old fry (*p* < 0.05). In the control sample, hatching weight was on average 0.09 ± 0.01 g. In contrast, in variants 2, 8 and 16 Se it was significantly higher, and it was 0.12 ± 0.01 g, 0.12 ± 0.01 g, and 0.13 ± 0.01 g, respectively (Figure 2b). The aforementioned fish parameters retained the initial tendency between the experimental groups. At the end of the experiment, the lowest length and weight of four-month-old fry were observed in the individuals from the control sample 36.17 ± 2.31 mm and 0.35 ± 0.10 g, respectively, and higher parameters were observed for 2, 8, 16 Se samples. In the 16 Se variant, an individual’s mean length and weight were 38.43 ± 3.55 mm and 0.44 ± 0.11 g (Figure 2a,b). A positive correlation between selenium accumulation and length and body weight of fry was observed (*p* < 0.05). The condition coefficient was similar between the experimental groups; in the four-month-old fry, it remained in the range between 0.72–0.75.

## 4. Discussion

Selenium is an essential nutrient for organisms but is toxic at high levels [1,2,12,14,26,27,28,29,30]. The results of our study show improved breeding of fish when the innovative method of supplementation of selenium to non-feeding stages of development was applied. Our research results confirm the key role of selenium in fish growth from the earliest stages of life [9,19,39,56,57,58]. Immersion of fertilised eggs of sea trout from the selenium-poor area (1.6 µg Se g-1 (dw) caused an increase in the amount of the element to the yolk then to the body of progeny. An increase in Se content to the desired level improved the growth of embryos. Se concentration in the hatch body for optimal growth for studied sea trout ranged from 4.5 to 5.3 µg g^−1^ (dw) compared to the control group (3.6 µg g^−1^, dw). The optimal selenium content in the body also had a slightly positive effect on reducing egg mortality and hatching rate (the effect was non-significant). These features also confirm the important role of selenium in fish condition and survival [12,14,19,21].

It is known that selenium overdose is harmful, and we observed a minor difference between the Se optimum and hazardous concentrations that occurred in the body [26,27,28,29,30]. In our study, the swelling of fertilised eggs in water supplemented with SeO_2_ at a concentration of 16 mg Se L^−1^ was associated with a significantly lower hatching rate when Se content in the hatch body was 5.4 µg Se g^−1^ (dw). Our results confirm that small differences between the appropriate and harmful concentrations of Se occurred [28,54]. A higher selenium concentration supplemented in our study resulted in the lethal effect of embryos at eyed-egg-stage (LT_100_). The experiment by Kowalska-Góralska [68] conducted on rainbow trout with a selenium concentration of 5.6 µg Se g^−1^ in the hatch body resulted in a slightly negative impact on the hatching rate. The threshold is similar to that obtained in the current study. Some authors indicated a somewhat lower threshold of >4 µg Se g^−1^ than mentioned by us above [32,35,75,76,77], but others have published a higher value. The EPA guideline toxicity threshold of selenium concentration for the whole-body tissue or muscle in fish was set at 8.5 and 11.3 µg g^−1^ (dw), respectively [40]. DeForest et al. [31] suggested a value of 9 µg g^−1^. The large differences in Se concentrations disclosed in papers are probably related to the age of the fish, their ability to bioaccumulate this element, and the species.

In wild fish from different world areas, Se concentration in the body is typically below 1 µg g^−1^ [52,73,78,79] or slightly above [73,78,79]. In cultured Atlantic salmon, 3.4 µg Se g^−1^ was recorded [74]. Se concentration in the whole body in polluted areas reaches more than 10 µg g^−1^ [80]. In contaminated water, particularly high selenium concentrations in fish, liver, kidney, eggs, muscles, and bones have been found [33,37,58,81]. The reference value (dw) for the selenium content in fish egg/ovary in the EPA guidelines is 15.1 µg g^−1^ [40]. Other authors indicate a value of 10 µg g^−1^ [27] or 17 µg g^−1^ [31]. Rudolph et al. [30] did not find body deformities of the *cutthroat trout* (*Oncorhynchus clarki lewisi*) fry when the concentration of Se in the eggs was 20.6 µg g^−1^ (dw). Our study observed singular cases (0.3%) of spine deformities at hatch from groups with additional selenium dosage. Whereas in areas polluted with selenium, spine malformation is often noticed [26,27,28,29,30]. A deteriorating effect for bone tissue caused by other higher trace elements concentrations was also reported [82,83], particularly when the contamination was of heavy metal [84,85].

## 5. Conclusions

In selenium-poor areas, immersion of fertilised eggs in water enriched with Se during egg swelling can constitute a method to supplement the element to non-feeding stages of fish. The innovative method can be implemented in aquaculture to improve the growth of progeny and possibly fish survival in nature when larger individuals are stocked into watercourses. The selenium applied also had a slightly positive effect (not statistically significant) on reducing egg mortality and increasing the hatching rate. Se concentration added to water should be adjusted to the selenium compound, fish species, and Se’s content in the yolk. Se concentration of 4–8 mg L^−1^ as SeO_2_ is recommended as optimal for practical application for studied sea trout.

## Figures and Tables

**Table 1:** Selenium content in sea trout (*Salmo trutta* m*. trutta*) hatch and fry growing from eggs exposed to different concentrations of inorganic selenium (SeO_2_) in water during egg swelling. One-way ANOVAs and then Fisher tests were used for the post-hoc comparison. Values marked with the same letter are not significantly different from one another (*p* > 0.05). Mean value ± SD.

Se in Water [mg L^−1^]	0	1	2	8	16
	Selenium concentration in fish body (dw) [µg·g^−1^]				
Hatch *	3.61 ± 0.21 ^b^	4.49 ± 0.62 ^c^	4.75 ± 0.32 ^cd^	5.31 ± 0.32 ^cd^	5.40 ± 0.34 ^d^
four-month-old fry **	2.74 ± 0.30 ^a^	2.96 ± 0.41 ^a^	3.70 ± 0.16 ^b^	4.24 ± 0.51 ^b^	4.94 ± 0.35 ^cd^

* selenium content in dry weight calculated from wet weight assuming 87.5% moisture of hatch after Elliott [72]. ** selenium content in dry weight calculated from wet weight assuming 75% moisture of fry after Abdulsahib et al. [73]; Atanasoff et al. [74].
